# Transcriptome and metabolome profiling of *Narcissus pseudonarcissus* ‘King Alfred’ reveal components of Amaryllidaceae alkaloid metabolism

**DOI:** 10.1038/s41598-017-17724-0

**Published:** 2017-12-11

**Authors:** Aparna Singh, Isabel Desgagné-Penix

**Affiliations:** 1 Department of Chemistry, Biochemistry and Physics, Université du Québec à Trois-Rivières, 3351 boul. des Forges, Trois-Rivières, QC G9A 5H7 Canada; 2 Plant Biology Research Group, Université du Québec à Trois-Rivières, 3351 boul. des Forges, Trois-Rivières, QC G9A 5H7 Canada

## Abstract

Amaryllidaceae alkaloids (AAs) represent a diverse class of plant specialized metabolites and many display potent pharmacological activities. The AA metabolic pathway is poorly understood and resources are minimal. To enable AA pathway elucidation and novel biosynthetic enzymes discovery, we generated comprehensive metabolomic and corresponding transcriptomic datasets from different tissues of *Narcissus pseudonarcissus ‘*King Alfred’. In this study, we performed untargeted UPLC-QTOF-MS metabolite analysis from different tissues, which generated exhaustive list of compounds, including several AAs, most predominant and diverse in bulbs. RNA sequencing of *N*. *pseudonarcissus ‘*King Alfred’ bulbs yielded 195,347 transcripts, after assembly. Top expressed genes belong to process like metabolism, survival, and defense including alkaloid biosynthetic genes. The transcriptome contained complete sequences for all proposed genes encoding AA-biosynthetic enzymes such as tyrosine decarboxylase (*TYDC1* and *TYDC2*), phenylalanine ammonia-lyase (*PAL1* and *PAL2*) and phenolic acids hydroxylases (*C4H* and *C3H*) to name a few. Furthermore, transcriptome data were validated using RT-qPCR analysis and expression study in different tissues of *N*. *pseudonarcissus* ‘King Alfred’ was performed. Here, we present the first comprehensive metabolome and transcriptome study from *N*. *pseudonarcissus* ‘King Alfred’ providing invaluable resources for metabolic engineering and biotechnological applications.

## Introduction

Plants produce a plethora of chemicals important for growth, development, and defense through the primary and specialized (*aka* secondary) metabolic pathways. The specialized metabolites, also named phytochemicals or natural products, are specific and restricted to some taxonomic groups and play pivotal roles in plant defense, protection, and survival^1^.

The medicinal properties of the Amaryllidaceae are owed to the presence of specialized metabolites, the Amaryllidaceae alkaloids (AAs), which are specific to this plant family. Several AAs display pharmaceutical activities such as the anti-cancer narciclasine, the anti-parasitic haemanthamine, the anti-viral lycorine and the anti-acetylcholine esterase galanthamine^2–9^. Despite AAs being a huge therapeutic reserve, galanthamine is the only one used medically to treat the symptoms of Alzheimer’s disease through its reversible acetylcholine esterase inhibition and its nicotinic receptor binding activity^10,11^. Currently, the knowledge of AA metabolism and regulation is limited and only a few genes encoding biosynthetic enzymes have been identified^12–14^. A better understanding of AA biosynthesis will allow for the development of new cultivars or biotechnologies to help produce these valued phytochemicals. Additionally, over 600 AAs have been identified^15^, adding to the diversity of AA chemical structures and also demonstrating the complexity of their biosynthetic pathway.

**Figure 1:**
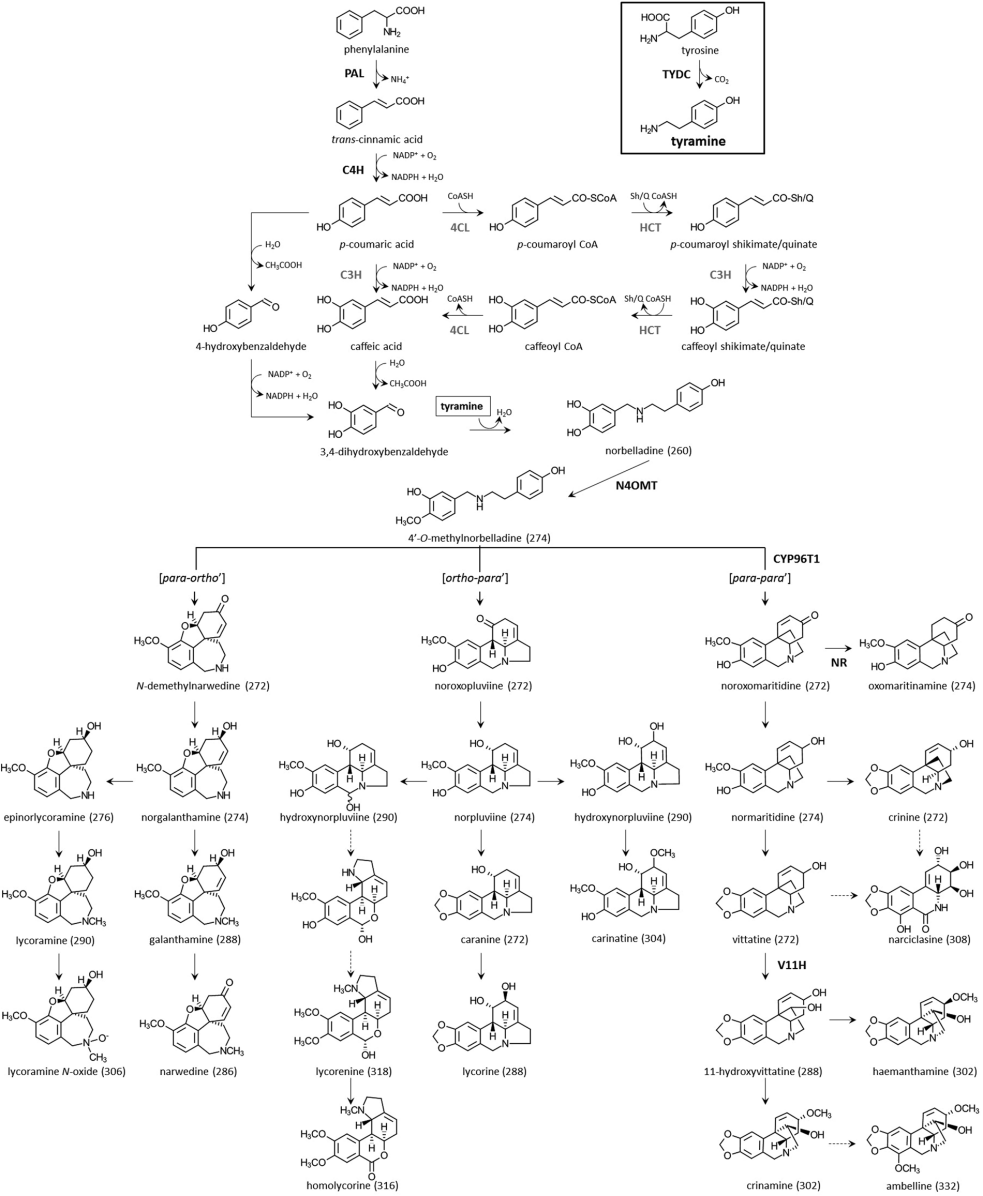
Proposed biosynthetic pathway leading to multiple Amaryllidaceae alkaloids. Enzymes for which corresponding genes have been isolated from Amaryllidaceae are shown in bold black whereas from other plants are shown in bold grey. Broken arrow represents more than one biochemical reaction. Number in parenthesis corresponds to molecular ion *m/z* of [M + H] of alkaloid in positive MS mode detection. Abbreviations: PAL, phenylalanine ammonia lyase; C4H, *trans-*cinnamate hydroxylase (CYP73A1); 4CL, 4-hydroxycinnamoyl CoA ligase; HCT, hydroxycinnamoyl transferase; C3H, *p-*coumarate hydroxylase (CYP98A3); TYDC, tyramine decarboxylase; N4OMT, norbelladine 4-*O*-methyltransferase; NR, noroxomaritidine reductase; V11H, vittatine 11-hydroxylase; Sh/Q, shikimate/quinate.

Previous studies using radiolabeled precursors led to the biochemical elucidation of the initial steps in AA biosynthesis^16–18^. Despite the vast structural diversity, all AAs are derived from a common central intermediate norbelladine, which is formed through the condensation of 3,4-dihydroxybenzaldehyde (3,4-DHBA) and tyramine (Fig. 1). Similarities to several plant specialized metabolic pathways can be observed. For example, tyrosine is decarboxylated to tyramine (Fig. 1). In opium poppy, this reaction is catalyzed by tyrosine decarboxylase (TYDC), a key regulating enzyme in the formation of isoquinoline alkaloids such as morphine and codeine^19–21^. The synthesis of 3,4-DHBA features steps similar to the phenylpropanoid pathway starting with the deamination of phenylalanine, a reaction catalyzed by phenylalanine ammonia-lyase (PAL) to generate *trans*-cinnamic acid (Fig. 1). *PAL* genes have been characterized from various species including Amaryllidaceae species^22,23^. Next, cytochrome P450-dependent monooxygenases (CYPs) namely cinnamate-4-hydroxylase (C4H) and coumarate-3-hydroxylase (C3H) may be involved to hydroxylate *trans-*cinnamic acid and *p*-coumaric acid, respectively. From there, several routes have been hypothesized to yield 3,4-DHBA (Fig. 1). However, it is still not clear whether the chain shortening reaction occurs on *p*-coumaric acid or caffeic acid^24–26^.

The first committed step in AA biosynthesis starts with the coupling of the two precursors, defining the entry point of primary metabolites into AA biosynthetic pathway^27–30^. The combination of 3,4-DHBA and tyramine results in the formation of a Schiff base intermediate, which following reduction, yields norbelladine. Similar condensation reactions are described in other plant alkaloid biosynthetic pathways. For example, the first committed step in the formation of benzylisoquinoline alkaloids is the combination of 4-hydroxyphehylacetaldehyde and dopamine catalyzed by norcoclaurine synthase (NCS) to produce norcoclaurine^31–33^. Next, norbelladine is methylated to 4′-*O*-methylnorbelladine by the norbelladine 4′-*O*-methyltransferase (N4OMT) which was identified in *Narcissus* sp. *aff*. *pseudonarcissus*, and enzymatically characterized by heterologous expression in *Escherichia coli* ^12^. The cyclization of 4′-*O*-normethylbelladine can occur by three different ways of intramolecular C-C oxidative phenol coupling named *para-ortho’*, *ortho-para’*, and *para-para*’ that generates three backbone structures (Fig. 1). The three AA backbones form the basis of further alkaloid diversity. A complex network of enzymatic reactions exists to produce a spectrum of compounds that differs between species, varieties, and cultivars and even between the different tissues of the same plant. These biochemical modifications are achieved by a multitude of enzymes catalyzing various types of reactions, such as C-C and C-O bond formations, *O*- and *N*-methylations, demethylations, hydroxylations, oxidations and reductions (Fig. 1).

Although a few genes involved in AA metabolism in *Narcissus* have been isolated, several genes have not yet been identified. Transcriptomic analysis coupled with metabolite profiling has become a popular tool for the discovery of novel genes encoding proteins involved in alkaloid biosynthesis^34–37^. Specifically, RNA sequencing using Illumina technology has been performed to analyze transcriptomes of various medicinal or nutritional plants, and the coupling with metabolomic analysis has greatly helped with the elucidation of several specialized metabolic pathways^38–41^. For example, metabolite and genome-wide transcript profiling collectively yielded known and previously undescribed gene transcripts and metabolites associated with the terpenoid-indole alkaloids in *Cathanranthus roseus* ^42^. In addition, comparing Illumina sequencing based transcriptome profile with metabolic data led to a better understanding of the biosynthesis of anti-cancerous alkaloids, camptothecin and anthraquinones^43^.

Although RNA sequencing has been performed on few species of Amaryllidaceae including *Lycoris aurea*, *Narcissus spp*., *Galanthus spp*., and *Leucojum aestivum* ^12,44,45^, AA metabolism still remains poorly understood. To enable pathway elucidation and novel enzyme discovery, we first performed metabolite profiling from different tissues of *N*. *pseudonarcissus* ‘King Alfred’ to identify the best tissues for RNA-seq. Leaves and bulbs showed high levels of AAs but since bulbs displayed more diversity, they were selected for transcriptomic analysis. Next, we created an extensive dataset for *N*. *pseudonarcissus* ‘King Alfred’. Using Illumina HiSeq. 2000 sequencing, we obtained a deeply sequenced transcriptome with 73,081,603 reads that were assembled into 195,347 transcripts. Comprehensive analysis of the transcriptome indicated the presence of previously characterized (*e*.*g*. *N4OMT*, *CYP96T1*, *NR*) and proposed (*e*.*g*. *C4H*, *C3H*, *HCT*, *4CL*) AA biosynthetic genes. We further validated our findings by performing expression analysis of each AA biosynthetic gene in different tissues of *N*. *pseudonarcissus* ‘King Alfred’ using RT-qPCR analysis. Used together, these unprecedented resources allow the assembly of a biochemical snapshot representing AA metabolism, guiding pathway elucidation and search efforts for new biosynthetic enzymes. Moreover, the availability of enzymes variants will expand the ‘toolbox’ essential to synthetic biology efforts^46^.

## Results

### Alkaloid profiling of *Narcissus pseudonarcissus* ‘King Alfred’

**Figure 2:**
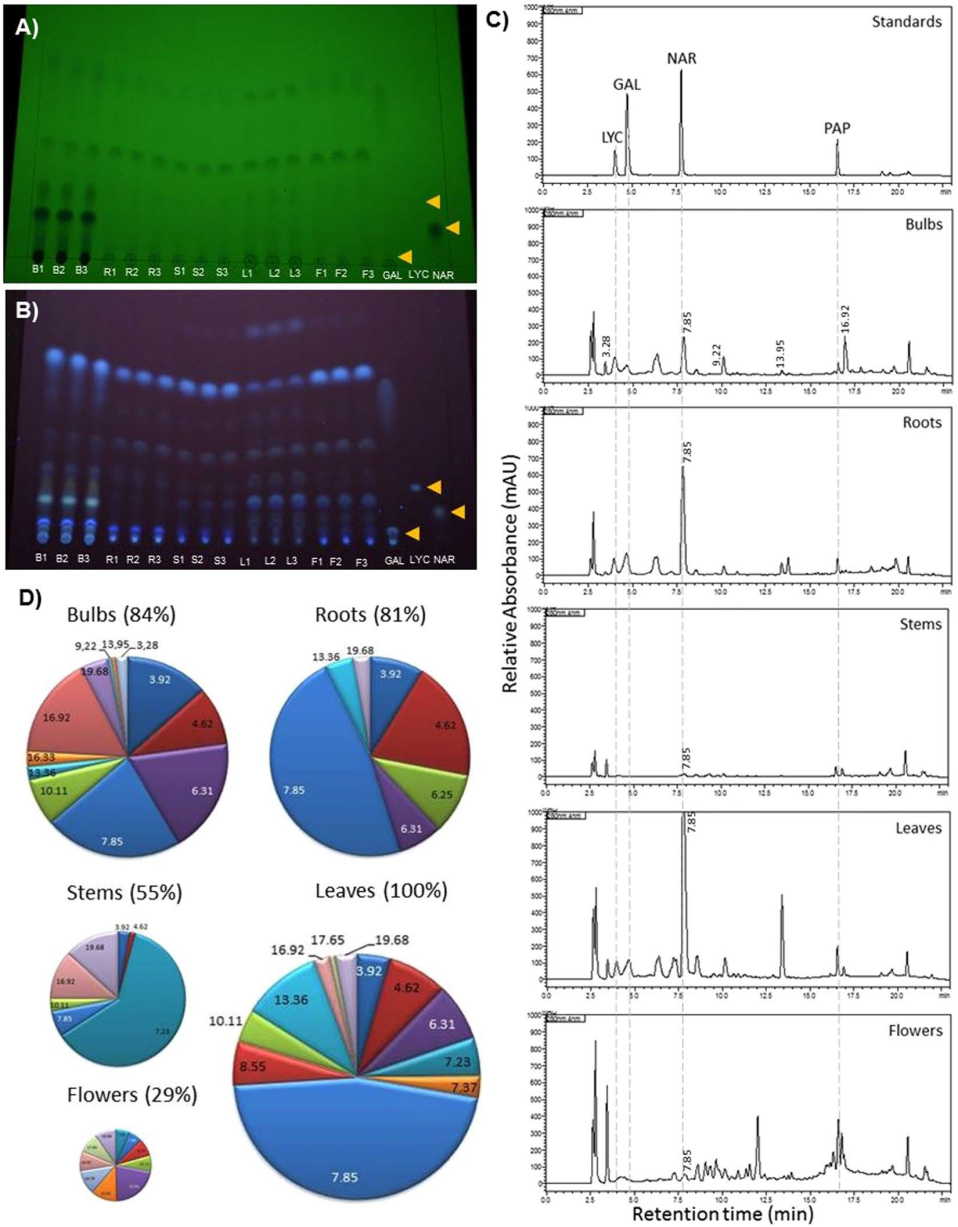
Chromatographic analysis of compounds obtained from an acid-base extraction of different tissues of *N*. *pseudonarcissus* King Alfred. (**A**,**B**) Thin-layer chromatography of extracts developed at 254 nm (**A**) and at 365 nm (**B**). (**C**) Representative HPLC chromatogram for known standards (top) such as lycorine (LYC), galanthamine (GAL), narciclasine (NAR) and papaverine (PAP) and from extracts of five different tissues of *N. pseudonarcissus* King Alfred. (**D**) Pie charts representing the relative abundance of major compounds in extracts of *N. pseudonarcissus* and normalized using the papaverine internal standard, determined by HPLC analysis. The number in each portion of the pie chart represents the retention time (Rt) of each compound.

An optimized acid-base extraction was used to separate alkaloids from other organic compounds based on their acid-base properties. Alkaloid profiles (composition and concentration) from different extracted tissues (bulbs, roots, stems, leaves, and flowers) of *N*. *pseudonarcissus* ‘King Alfred’ were initially screened using chromatographic methods (Fig. 2). Thin-layer chromatography (TLC) showed qualitative differences among alkaloids from different tissues (Fig. 2A,B). We found that all plant parts contain various known and unknown compounds. Bulbs possess a larger number of diverse alkaloids, including large spots on TLC with R_f_ corresponding to galanthamine (R_f_ 0.02) and lycorine (R_f_ 0.13) standards but not to narciclasine (R_f_ 0.08) (Fig. 2A,B). Stem extracts appear to contain fewer alkaloids than the other tissues. Roots, leaves and flowers also contain spots with R_f_ corresponding to galanthamine and lycorine (Fig. 2A,B). Spots with Rf corresponding to narciclasine were not detected in any plant samples. Thus, TLC analyses showed strong differences in the alkaloid profiles of the extracts from different tissues of *N*. *pseudonarcissus* ‘King Alfred’.

To quantify the differences in alkaloid profiles, high-performance liquid chromatography (HPLC) with a photodiode array (PDA) detector analyses were performed. HPLC-PDA chromatograms confirmed differences among the extracts from different tissues. Peaks were detected at similar retention times (R_t_) as those of AA standards lycorine (R_t_ 3.92 min), galanthamine (R_t_ 4.62 min) and narciclasine (R_t_ 7.75 min). For example, peaks for lycorine and galanthamine were detected in bulbs, roots and leaves (Fig. 2C), although in some case the absorption spectra were slightly different than those of standards. Also, peaks corresponding to narciclasine (R_t_ and absorption spectra) were not detected in any tissue tested, which correlates with TLC data. Interestingly, an unknown compound (R_t_ 7.85 min) was present in all tissue tested and was most abundant in leaves whereas compounds at R_t_ 6.31 and 16.92 min were two times more abundant in bulbs compared to other tissues (Fig. 2C-D). In addition, compounds at R_t_ 3.28, 9.22 and 13.95 min were only detected in bulbs, suggesting specificity for this tissue. Relative quantification of the alkaloid content of extracts from different tissues was done after normalization using papaverine an the internal standard. Total AA levels were highest in leaves and were normalized to 100%, followed by bulbs (84%), roots (81%) and stems (55%) (Fig. 2D). Flowers (29%) contained low alkaloid levels. Altogether, the results show different alkaloid profiles for the different tissues of *N*. *pseudonarcissus* ‘King Alfred’ with leaves containing the highest level of compounds followed by bulbs, each having high levels of numerous specific compounds.

### Metabolite profiling by LC-MS

Untargeted UPLC-QTOF-MS profiling was performed to generate extensive mass lists corresponding to a wide variety of metabolites, including alkaloids present in our various extracts of *N*. *pseudonarcissus* ‘King Alfred’ tissues. Less overall number of ion masses were detected in analysis performed in negative ion mode analysis, between 4 and 12 (Table 1), with 4-hydroxybenzyldehyde was the most abundant compound present in bulbs and roots (Supplementary file 1). Analysis performed in positive ion mode detected between 55 and 71 compounds including several alkaloids, fatty acids, organic acids, and sugars (Table 1, Supplementary file 1). AAs detected using UPLC-QTOF-MS analyses in positive mode were extracted and compiled in Table 2 to identify and expand our targeted AA profile of our different extracts of *N*. *pseudonarcissus* ‘King Alfred’. It must be noted that some metabolites are being listed twice with slightly different retention times or ion masses. There are several reasons explaining this. First, the identification provided is a proposed identification. For the same AA compound, different structural isomers may be present and separate on the column to yield different retention times. For slightly different Rt it may be epimers whereas larger Rt differences may indicate enantiomers. For example, crinamine [M + H] is a C-3 methoxy epimer of haemanthamine, thus sharing the same [M + H]. Another example related to the epimers vittatine and crinine also with the same masses. Secondly, the MZmine software identifies peaks via searches through custom and online database. Alkaloids are present but only few AAs are available. Thus, the potential identification of AA using these databases is lower than for well-known metabolites.

**Table 1:** Total number of ion masses identified by UPLC-QTOF-MS analysis of extracts of *N. pseudonarcissus* ‘King Alfred’ tissues using positive and negative ionization mode.

*N. pseudonarcissus* ‘King Alfred’ tissue extracted	Number of ion masses	
	Positive ion mode (M + H)	Negative ion mode (M − H)
Bulbs	71	12
Roots	61	10
Stems	58	7
Leaves	71	5
Flowers	55	4

**Table 2:**
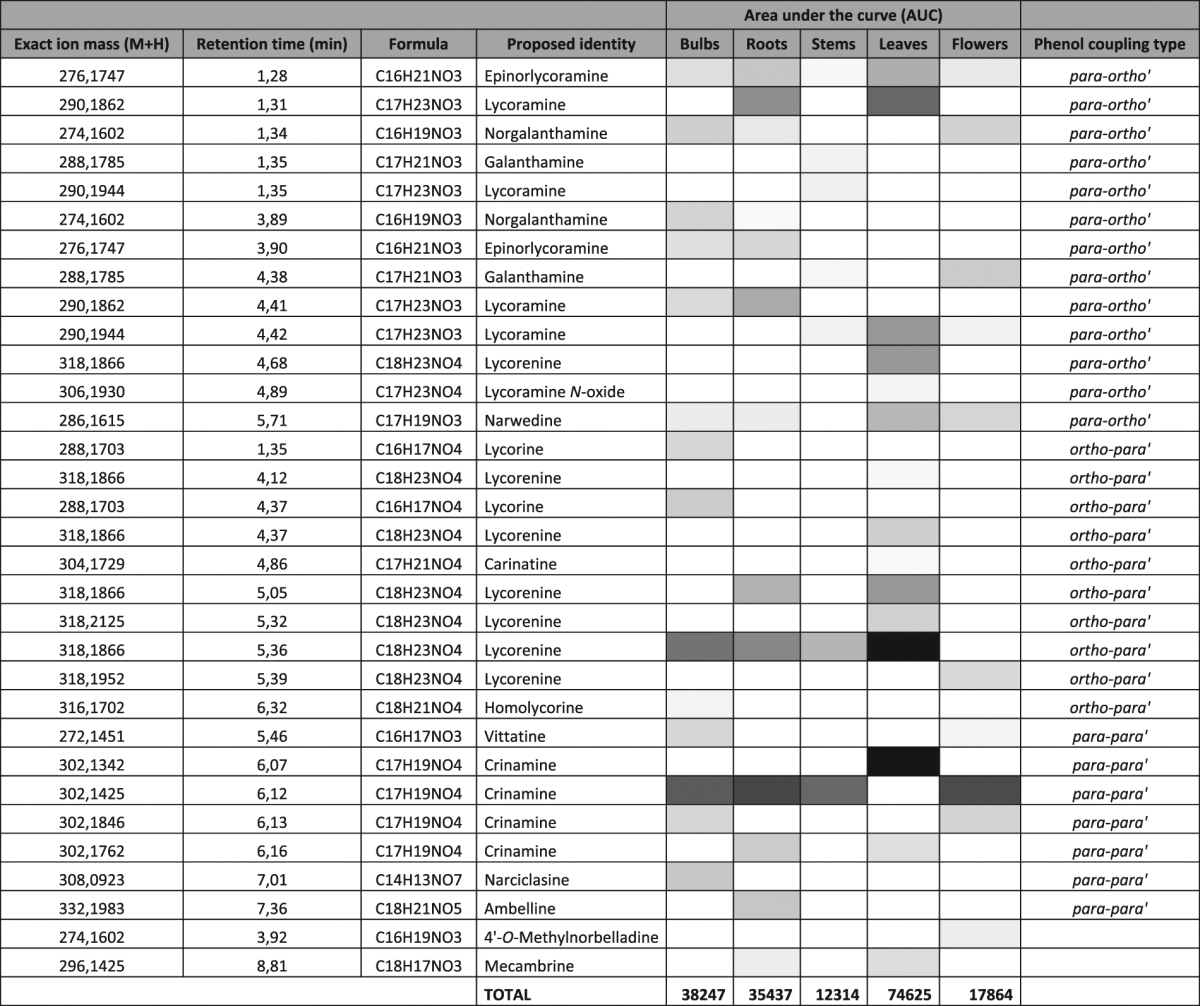
Amaryllidaceae alkaloids detected by UPLC-QTOF-MS and the relative abundance detected through the area under de curve. The accurate quantitative corresponding values of the area under the curve are listed in Supplementary file 1.

The most abundant AA detected was crinamine (R_t_ 6.07; [M + H] 302.1341) (Table 2, Supplementary file 1), followed by lycorenine (R_t_ 5.36; [M + H] 318.1866) in leaves. The most abundant AA in flower, stem, root and bulb tissues was crinamine (R_t_ 6.12, [M + H] 302.1425). Lycorine and homolycorine were only detected in bulbs whereas epinorlycoramine was detected in all tissues (Table 2, Supplementary file 1). Interestingly, 4′-*O*-methylnorbelladine was only detected in flower tissues.

### cDNA library, Illumina sequencing, *de novo* assembly, and annotation

To have a better understanding of the AA biosynthetic pathway and to enable the discovery of genes involved in their formation, we performed RNA sequencing of *N*. *pseudonarcissus ‘*King Alfred’. Bulb tissues were chosen for transcriptomic analysis primarily based on alkaloid accumulation profiles determined above. RNA was extracted and screened for sufficient quality and quantity prior to cDNA library creation and deep sequencing using Illumina. A total of 73,081,603 raw paired reads were obtained, which were trimmed to give 66,054,792 surviving paired reads that corresponds to 90.4% (Table 3). For non-model plants such as Amaryllidaceae, whose genomic information is lacking, a *de novo* assembly is necessary. Detailed overview of the sequencing output, trimming, normalization, *de novo* assembly, and transcript length distribution graphs is provided in Supplementary File 2. A total of 10,523,399 pair reads were obtained after the normalization step corresponding to 15.93%. Normalized reads were used to assemble the transcriptome generating 195,347 transcripts (or unigenes).

**Table 3:** Summary of the transcriptome database generated from one lane of Illumina sequencing of *N. pseudonarcissus* ‘King Alfred’ bulbs.

*N. pseudonarcissus* ‘King Alfred’	
Total number of raw paired reads^a^	73 081 603
Total number of surviving paired reads trimmed^b^	66 054 792
Total number of surviving paired reads normalized^c^	10 523 999
Number of transcripts^d^	195 347
Number of components^e^	98 332
Number of annotated transcripts^f^	11 708

^a^Number of Paired Reads obtained from the sequencer.

^b^Number of Remaining Paired Reads after the trimming step.

^c^Number of Remaining Paired Reads after the normalization step.

^d^Number of transcripts:Trinity has created a list of transcripts representing the transcriptome isoforms.

^e^The transcripts are grouped in components loosely representing genes. Transcript names are prefixed by the component/gene name e.g. transcripts c115_g5_i1 and c115_g5_i2 are derived from the same isolated de Bruijn graph and therefore share the same component/gene number c115_g5.

^f^Each transcript has been aligned against the *uniprot_sprot.trinotate_v2.0.pep* protein database using blastx program from the NCBI BLAST family.

For identification, the assembled transcripts were aligned against the uniprot_sprot.trinotate_v2.0.pep protein database. BLAST annotation yielded 11,708 transcripts of an average length of 1,463 bp (Supplementary File 2). Thus, a large number of transcripts showed no similarity to known genes and appears to represent transcripts of uncharacterized genes or sequences specific to *N*. *pseudonarcissus* ‘King Alfred’. Altogether, we concluded that a good quality transcriptome was developed with a high number of surviving pair reads and assembled transcripts.

The 125 most abundant transcripts present in the transcriptome of *N*. *pseudonarcissus ‘*King Alfred’ bulbs were identified and compiled in Supplementary file 3 and represent approximately 16% of the transcriptome. First, it is interesting to note that bulb tissues contain viral RNA and the top four expressed transcripts belong to the *Narcissus* mosaic virus although no symptoms were detected. The top expressed plant genes belong to a group of plant survival and defense genes such as metallothionein-like protein, catalase, and peroxidases that help plants to defend against pathogens such as viruses. The second most highly represented category was primary metabolism including sucrose synthase, fructosyltransferase, glyceraldehyde 3-phosphate dehydrogenase, enolase, starch synthase, mannose-1-phosphate guanylyl transferase, UDP glucoronate 4-epimerase, *S-*adenosylhomocysteinase, and ATP synthase. Other highly represented categories were structural and transport proteins such as tubulin, aquaporin, transmembrane protein, BAC transporter, GRP2A, cathepsin, ADP ribosylation, and ADP/ATP carrier protein. Altogether, most abundant transcripts represent important sets of genes involved in cellular and metabolic process such as plant defense, primary metabolism, and structural and transport genes. Three transcripts encoding alkaloid biosynthetic genes, *S-norcoclaurine synthase1-like* (*NCS*; rank 62), *vittatine 11-hydroxylase* (rank 98) and *N4OMT3* (rank 125), were detected among the most abundantly expressed transcripts (Supplementary file 3). NCS catalyzes the first committed step in benzylisoquinoline alkaloid biosynthesis which involves a stereoselective Pictet-Spengler condensation of dopamine and 4-hydroxyphenylacetaldehyde to form (*S*)-norcoclaurine^31–33^.

**Figure 3:**
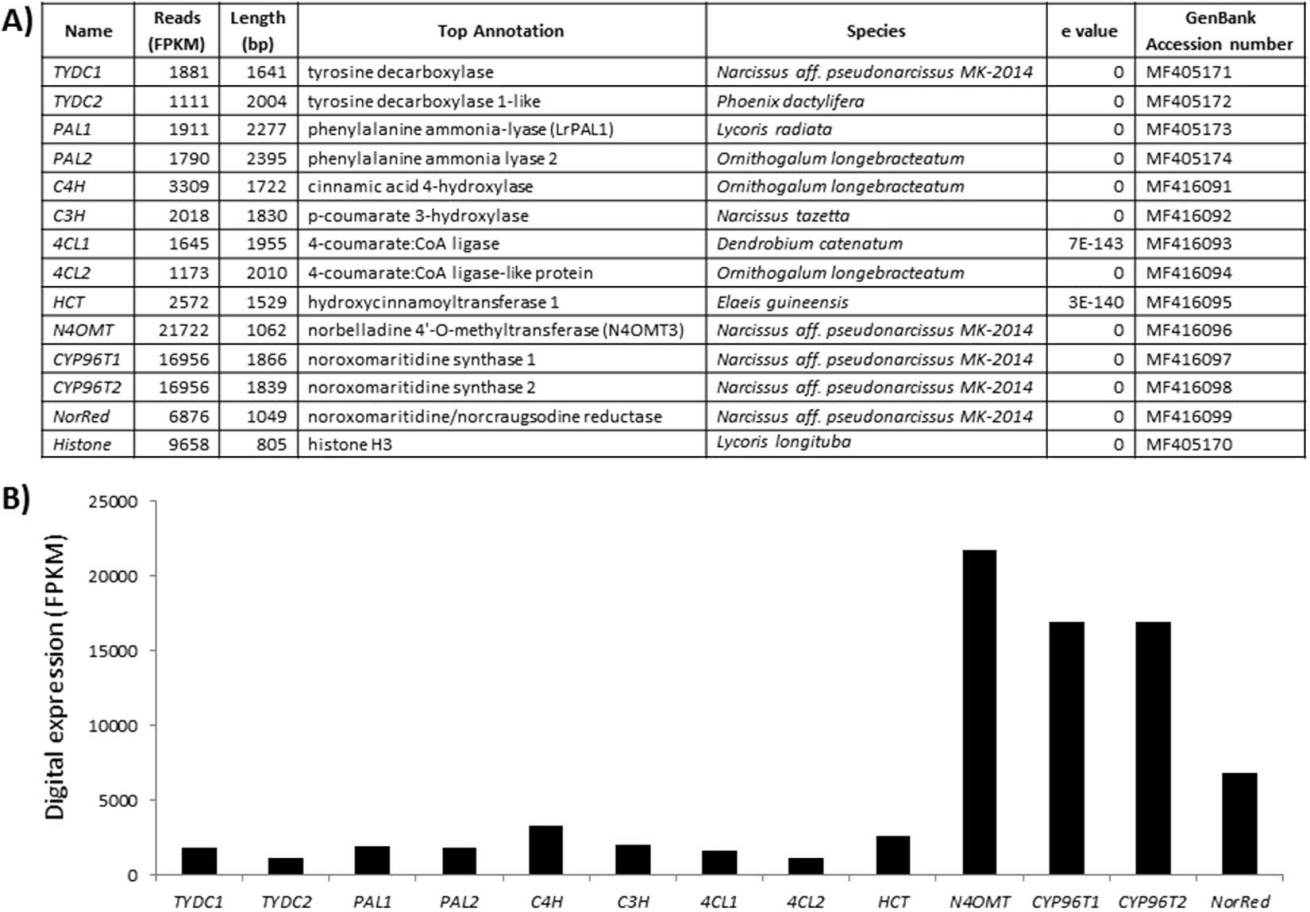
Summary of the biosynthetic genes transcripts corresponding to enzymes involved in Amaryllidaceae alkaloids metabolism in the bulbs transcriptome of *N. pseudonarcissus* King Alfred. (**A**) Table listing AA biosynthetic gene transcripts identified with top BLASTx annotation, corresponding e-value and GenBank accession number. All genes reported in this table correspond to full-length transcript. (**B**) Digital expression of AA biosynthetic genes expressed in fragments per kilobase million (FPKM).

Local BLASTx analyses were performed to identify specific gene transcripts encoding enzymes putatively involved in alkaloid biosynthesis (Fig. 3A). From the precursor pathway leading to norbelladine (Fig. 1), several transcript isoforms of orthologous genes were identified. For example, *PAL* genes have been cloned and characterized from various plant species including the Amaryllidaceae *Lycoris radiata* where two isoforms (*LrPAL1* and *LrPAL2*) were identified^22,23^. BLASTx searches for *PAL* genes in the transcriptome led to the identification of 19 transcripts (5 full-length) from 6 different components. A component is a family of similar transcripts. For example, component ‘contig1’ may include several similar transcript sequences (contig1A, contig1B, contig1C, etc.) most likely RNA variants or isoforms of the same family. Of those, only two transcripts from 2 different components were >98% similar to *PAL* sequence. Thus, we identified two full-length *PAL* isoforms identified as *NpPAL1* and *NpPAL2*. *NpPAL1* was most similar to *LrPAL1* and *NpPAL2* isoforms annotated closer to *PAL* from *Ornithogalum longebracteatum* (sea onion) with an E-value of zero (Fig. 3A). Similarly, 14 gene transcripts (2 components) were found for *TYDC* but only two full-length isoforms were identified. For gene transcripts specific to AA biosynthesis, we were able to identify all three genes with one isoform of *N4OMT*, two isoforms of *CYP96Ts* (*CYP96T1* and *CYP96T2*) and one isoform of *NorRed* (Fig. 3A). Thus, we were able to find full-length transcripts and several isoforms with E-values corresponding to genes encoding enzymes involved in AA biosynthesis.

In the central AA biosynthetic pathway, from norbelladine to oxomaritidine (Fig. 1), sequence reads corresponding to *N4OMT3*, cytochrome P450s (*CYP96T1* and *CYP96T2*), and *NorRed* were more abundant than those operating in precursor pathways such as *PAL*, *TYDC*, and others. This was observed using comparative fragments per kilobase million (FPKM) digital expression (Fig. 3B). FPKM normalizes for sequencing depth and gene length. From the precursor pathway leading to norbelladine (Fig. 1), most transcripts were expressed at similar levels suggesting coordinated regulation for the formation of AA precursors.

### Gene expression validation with RT-qPCR

**Figure 4:**
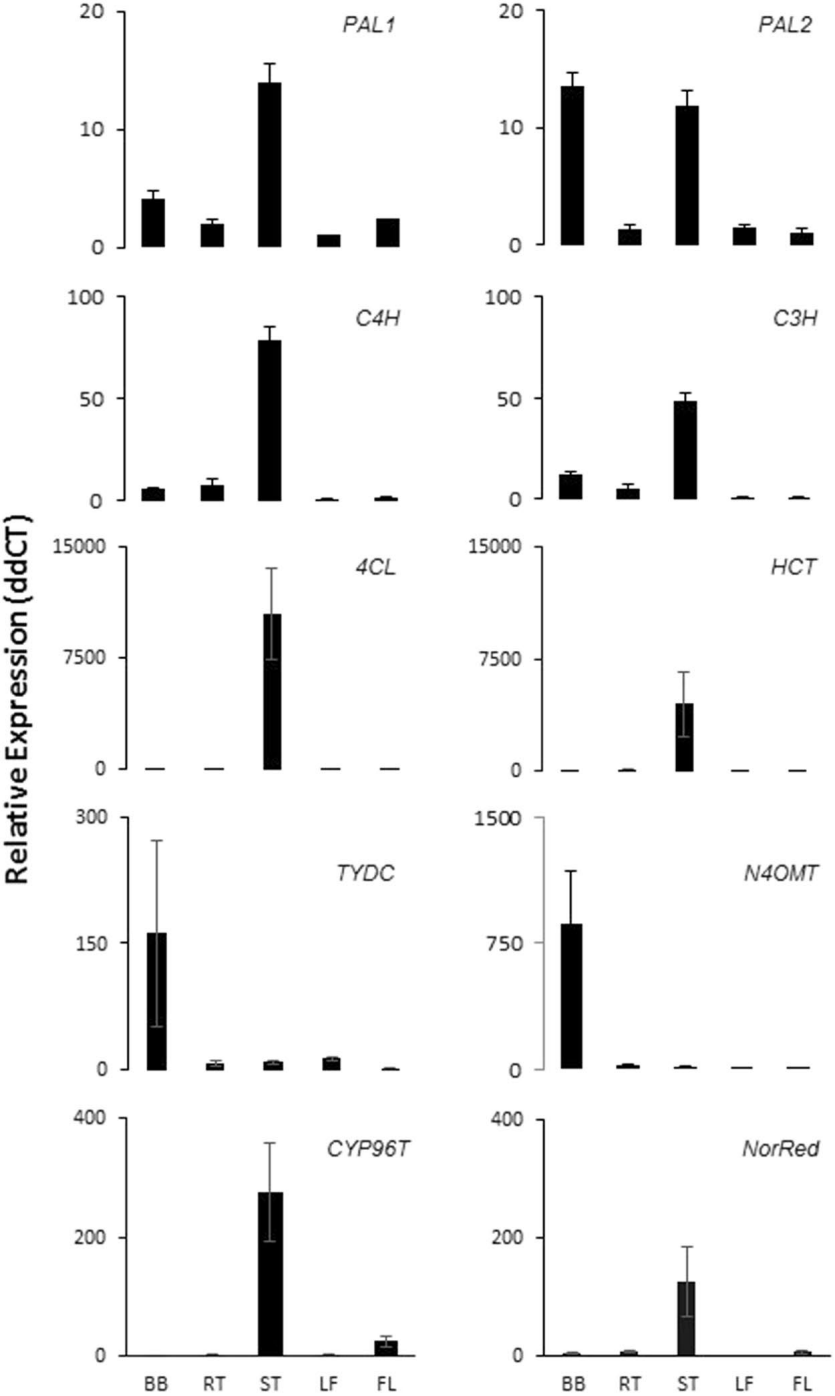
Quantitative real-time PCR results of ten AAs biosynthesis pathway genes in different tissues of *N. pseudonarcissus* ‘King Alfred’. Graphs are plotted using normalized ddCT values scaled to lowest. Histone was used for internal reference. Expression fold change and error bars were calculated using the comparative 2^−ΔΔCt^ method^72^ from three independent experiments. Bars represent the mean standard deviation of three independent replicates. Abbreviations: Gene studied are *PAL*, *phenylalanine ammonia lyase; C4H, trans-cinnamate hydroxylase; C3H, p-coumarate hydroxylase; 4CL, 4-hydroxycinnamoyl CoA ligase; HCT, hydroxycinnamoyl transferase; TYDC, tyramine decarboxylase; N4OMT, norbelladine 4-O-methyltransferase; CYP96T, noroxomaritidine synthase* and *NR, noroxomaritidine reductase*. Tissues of *N. pseudonarcissus* ‘King Alfred’ are BB, bulb; RT, root; ST, stem; LF, leaf; FL, flower.

To validate the gene expression data from the bulb transcriptome of *N*. *pseudonarcissus ‘*King Alfred’ and to assess transcript expression profiles across tissues, we performed reverse transcription-quantitative real-time PCR (RT-qPCR) analysis. All of the proposed AA biosynthetic gene transcripts were analyzed for expression profiles in different plant tissues (bulbs, roots, stems, leaves, and flowers) (Fig. 4). Only *N4OMT*, *TYDC*, and *PAL2* transcripts showed overexpression in bulbs compared to other tissues. Most phenylpropanoid precursor pathway genes (*PAL1*, *C4H*, *C3H*, *4CL*, and *HCT*), displayed a similar expression pattern; relatively high in stems followed by bulbs and low in other tissues (Fig. 4). Interestingly, *PAL1* and *PAL2* showed similar levels of expression in stems but *PAL2* expression was higher in bulbs than *PAL1*. From the central pathway, *N4OMT* was relatively the most highly expressed gene in bulb tissues (Fig. 4). AA specific genes *CYP96T* and *NorRed* were highly expressed in stems compared to other tissues, which did not correlate with *N4OMT* expression (Fig. 4). For most of the genes studied, leaves and flowers displayed the lowest expression compared to all other tissues. Taken together, the RT-qPCR results reflect relative differential gene expression profiles in different tissues.

### Integration of transcriptome data

**Figure 5:**
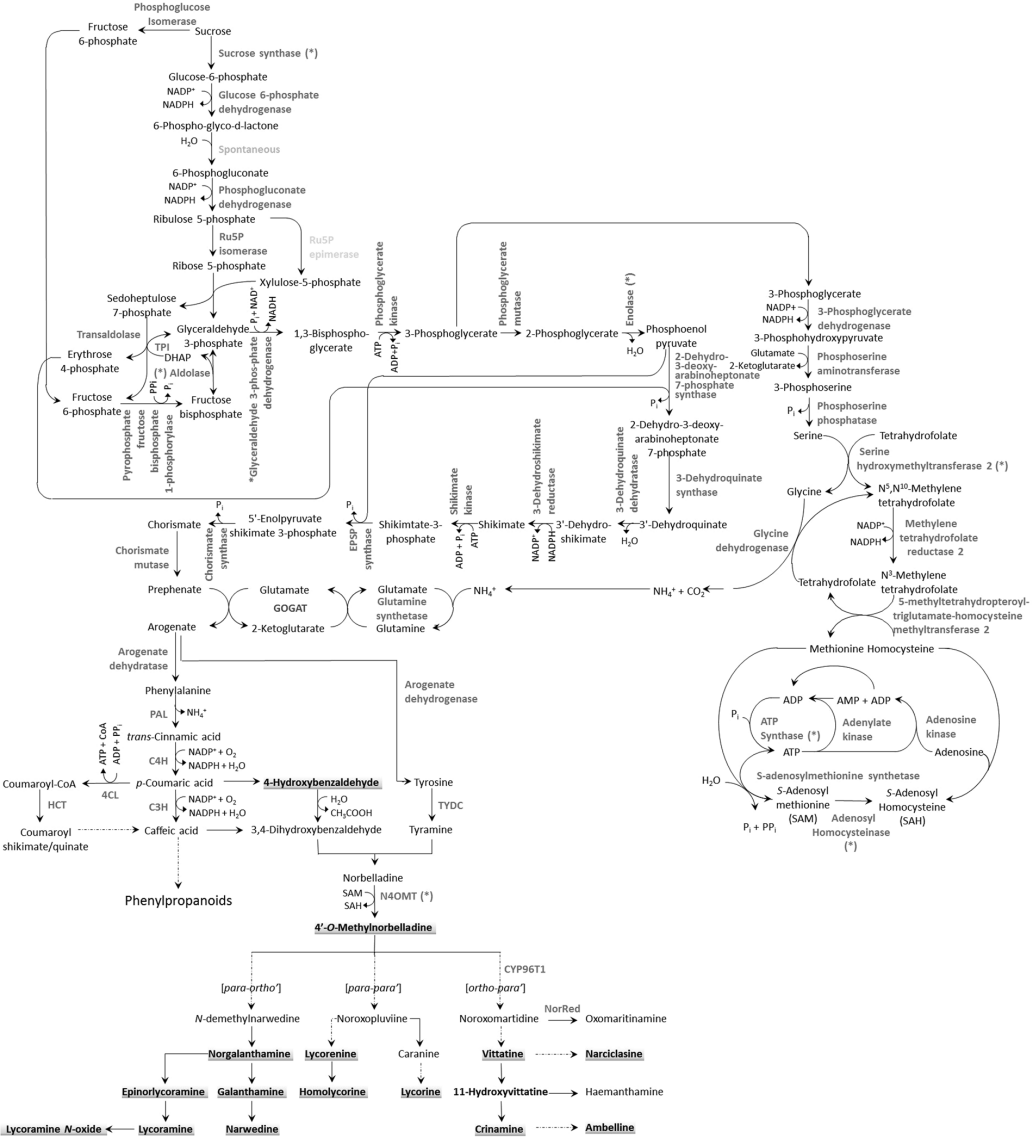
Metabolic networks from sucrose to Amaryllidaceae alkaloids. Gene transcripts corresponding to enzymes shown in dark grey next to arrow were identified in the transcriptome database. [Only Ru5P epimerase written in pale grey was not]. The (*) next to the enzyme name indicates gene transcripts present among the top 125 most abundant genes. Metabolites identified by UPLC-QTOF-MS are highlighted in bold black.

A broad survey of cellular metabolism involved in the conversion of sucrose to AAs resulted in the identification of transcripts and metabolites corresponding to a substantial number of metabolic steps (Fig. 5). With the exception of ribulose 5-phosphate epimerase (Ru5P epimerase), all enzymes required for the formation of AA precursors, tyramine and 3,4-DHBA were represented in the transcriptome database, whereas 14 AAs were found in the UPLC-QTOF-MS generated metabolite database (Supplementary file 1). Many of the transcripts encoding primary metabolic enzymes were also among the most abundant transcripts (Supplementary file 3). Notably, three independent gene transcripts in the top 125 encoded enzymes involved in the metabolism of *S*-adenosyl methionine (SAM). SAM is a common cosubstrate providing the methyl donor for the various reactions of *O*- and *N*-methylation in alkaloid biosynthesis. For example, the enzymes involved in the *N*-methylation of epinorlycoramine to lycoramine or the *O*-methylation of 11-hydroxyvittatine to crinamine could use SAM as the methyl donor (Fig. 1). Transcripts encoding enzymes proposed to be involved in AA precursor (tyramine and 3,4-DHBA) biosynthesis were identified along with the 4-hydroxybenzaldehyde metabolite. In addition, the three known AA specific gene transcripts *N4OMT*, *CYP96T* and *NorRed* were all identified in the transcriptome. However, AA metabolites from the oxomaritinamine branch pathway were not present in the QTOF-MS dataset.

The remaining AA biosynthetic enzymes, for which cognate cDNAs have not been isolated, catalyze the missing reactions. Those include the (1) chain-shortening steps involved in the formation of 3,4-DHBA, (2) the condensation of precursors to form norbelladine, and 3) the conversions to multiple AA intermediates (Figs 1 and 5). Some of these enzymes likely belong to known protein families including the CYPs, *O*- and *N*-methyltransferases, reductases, norcoclaurine synthase, dioxygenases, and oxidoreductases. Candidate transcripts with substantial identity to these protein families and other enzyme categories potentially involved in AA metabolism were found in the Illumina-generated transcript database. Thus, through comparative transcriptomics and metabolomics approach, we were able to detect several AAs and biosynthetic genes pointing towards high quality and thorough transcriptome that could be very useful for further research on missing genes of AAs pathway.

## Discussion

Integration of UPLC-QTOF-MS metabolite profiling and Illumina RNA sequencing was used to survey the metabolome and the transcriptome of *N*. *pseudonarcissus* ‘King Alfred’ tissues. The depth of each database provides new insights into AA metabolism and establishes valuable resources for the discovery of new genes involved in alkaloid metabolism.

Different alkaloid profiles were observed within the different tissues of *N*. *pseudonarcissus* ‘King Alfred’ with higher concentration of AA in leaves but higher diversity in bulbs (Fig. 2, Table 1, Supplementary file 1). These results relate to previously published data for on *N*. *pseudonarcissus* ‘Carlton’ and *N*. *confusus* where highest amount of AAs occurred in bulbs^47–51^. Our QTOF-MS data showed predominantly *ortho-para’* (*i*.*e*. lycorine-type) AAs in leaves whereas *para-ortho’* (*i*.*e*. galanthamine-type) and *papa-para’* (*i*.*e*. crinamine-type) were abundant in all tissues. These results correlate with GC-MS analysis of six ornamental species of *Narcissus* ^52^. This suggests that different biosynthetic routes might be active within tissues. Transport of alkaloid precursors, intermediates or end-products might also be possible between tissues.

RNA-Seq is a useful tool to generate resources for non-model plants with restricted genomic information. Transcriptome assembly yielded 195,347 transcripts with a mean length of 761 base pairs. Similar numbers, 106,450 transcripts of mean length of 551 bp, were found in the transcriptome of *Narcissus spp*.^12^, supporting the quality and the depth of the transcriptome. The four most abundant transcripts from the *N*. *pseudonarcissus* ‘King Alfred’ transcriptome belong to viruses (Supplementary file 3), suggesting a contamination of plant material. In horticulture, ornamental plants such as Narcissus species are usually propagated vegetatively which makes them more susceptible to an accumulation of viruses^53^. Virus infections are fairly common in Amaryllidaceae and up to 25 viruses have been reported^54,55^. Mosaic viruses are the most common and several methods to detect them have been developed^56^.

The ‘plants’ most abundant transcripts encoded proteins associated with metabolism, defense, transport and cellular structure similar to other plants^57^. For example, comparison of the most abundant transcripts of Amaryllidaceae bulbs with opium poppy revealed a similar pattern of expression, demonstrating that the genes involved in the common function of plant growth, development and defense are expressed at relatively consistent levels^34^. Also, transcripts encoding biosynthetic enzymes involved in the regeneration of (*S*)-adenosyl-methionine (Fig. 5; Supplementary file 3) were among the most abundant in the database, which is in agreement with abundant genes found in the transcriptome of other alkaloid-producing plants^34,58^. A *NCS-like* sequence was found among the top most expressed genes, suggesting its plausible role in AAs biosynthesis at the norbelladine formation step. Similar results were observed in opium poppy cell cultures^58^. Moreover, at rank 115 we detected a putative cysteine proteinase similar to the C3 chain-shortening enzyme from *V*. *planifolia* ^24–26^ capable of generating precursors 4-HBS or 3,4-DHBA. Interestingly, the presence of *vittatine 11-hydroxylase* among the top expressed genes correlates with elevated levels of crinamine (Supplementary file 3; Table 3).

FPKM is directly proportional to the abundance of specific transcript in the transcriptome, thus quantification of the data provided an accurate measure of relative expression through sequencing depth and gene length. From the precursor pathway leading to norbelladine (Fig. 1), most gene transcripts were expressed at similar levels suggesting coordinated regulation of the formation of AA precursors in bulbs (Fig. 3). Among the AA specific transcripts, *N4OMT3* and cytochrome P450s (*CYP96T1* and *CYP96T2*) were the most highly expressed ones, suggesting that they play crucial roles in bulb metabolic processes and that the AA central biosynthetic pathway expression is important in this tissue. Alkaloids are well known for their metabolic effects in animals (*e*.*g*. caffeine, nicotine, morphine and cocaine), and have probably evolved as defense compounds against herbivores and pathogens^59^. It was reported that the high alkaloid content of bulbs and leaves of Amaryllidaceae, primarily serves to protect the plant’s carbohydrate resources from herbivores and microbial organisms^60,61^. *PAL1* and *PAL2* expression was similar in bulbs, which correlates with previously reported *LrPAL1* and *LrPAL2* expression^23^. The presence of all proposed AA biosynthetic genes and the high levels of AA-specific genes, from the central pathway, correlates with the presence and high levels of AA in bulbs.

Comparison between digital expression (FPKM) and qPCR analysis from bulbs showed similar expression patterns for most genes (Figs 3B and 4). This was the case for *N4OMT*, which was highly expressed in both cases, and present among the most abundant transcripts (Supplementary file 3). *N4OMT* expression was followed by *CYP96T1* and *NR*. Also RT-qPCR analysis confirmed the expression of all AA biosynthetic genes found in the transcriptome and revealed several novel gene transcripts in the Illumina-generated transcriptome. *PAL1* and *PAL2* showed similar levels of expression in stems but *PAL2* expression was higher in bulbs than *PAL1* suggesting different regulations/roles for each isoform/enzyme variant.

Altogether, qPCR results reflect relative differential gene expression profiles in different tissues. Since phenylpropanoids are widely distributed throughout plants^62^, it is expected that the expression of genes encoding phenylpropanoid enzymes (*e*.*g*. *PAL, C4H, C3H, 4CL*, and *HCT*) do not necessarily correlate with alkaloid content. The higher expression of *CYP96T* and *NorRed* in tissues containing the less amount of AAs (*e*.*g*. stems) is puzzling. However AAs derived from these reactions were not detected in any of the *N*. *pseudonarcissus* ‘King Alfred’ tissues. Only *TYDC* and *N4OMT*’s high expression levels, correlated with high alkaloid content in bulbs, support a relation between high alkaloid content and AA biosynthetic gene expression. It is also possible that transport of enzymes and AAs occurs within plants. Such transport has previously been observed and described for benzylisoquinoline alkaloids^63–65^.

In conclusion, we generated the first comprehensive metabolome and transcriptome databases from *N*. *pseudonarcissus* ‘King Alfred’ using UPLC-QTOF-MS and next-generation Illumina sequencing. We identified diverse AA profiles in different tissues and confirmed the expression of proposed AA biosynthetic genes. Since the integration of such databases, metabolome and transcriptome, allows for the discovery of novel genes, this study not only increases the knowledge of AA metabolism but also provides tools for metabolic engineering. Indeed, a deeper understanding of the molecular mechanism involved in AA biosynthesis is crucial to take advantage of new biotechnologies for the production of a class of health-relevant alkaloids.

## Materials and Methods

### Plant material and chemicals

Ornamental bulbs of *N*. *pseudonarcissus* cultivar ‘King Alfred’ were purchased from Fraser’s Thimble farms (BC, Canada). Bulbs were planted in September in well-drained soil to grow until the flowering stage in the spring. Different plant tissues such as bulbs, roots, stems, leaves and flowers were harvested in May for metabolic and transcriptomic analyses. HPLC grade acetonitrile and methanol were purchased from Fisher Scientific (https://www.fishersci.com). Standards narciclasine and galantamine were purchased from Tocris Bioscience (Bristol, U.K.) whereas lycorine and papaverine was purchased from Sigma-Aldrich (ON, Canada).

### Alkaloid extraction and HPLC analysis

*N*. *pseudonarcissus* ‘King Alfred’ plant tissues (2 g/each in triplicate; bulbs, roots, stems, leaves and flowers) were crushed in liquid nitrogen and extracted with methanol (10 ml) for 24 hrs at RT on a shaker (200 rpm). Extracts were centrifuged at 12,000 rpm for 5 minutes to collect supernatant without debris and left for complete evaporation. The raw alkaloids obtained were further subjected to a modified acid-base extraction method (specific for alkaloids), where they were resuspended in methanol 100% pH-8 (adjusted with NH3) and H2SO4 (2% v/v). Organic impurities were removed by washing twice with chloroform. Alkalization was done using NH3. The purified alkaloid extracts obtained were dried under N2 gas and finally solubilized in 300 μL methanol. Qualitative analysis of alkaloids was performed on TLC silica gel 60 F_254_ aluminum sheets 20 × 20 cm, (Merck, Darmstadt, Germany). Ten μl of 100 ppm for each standard (GAL, LYC, NAR) and 15 μl of samples were loaded in triplicates on TLC and visualized under 280 nm and 365 nm. For qualitative and quantitative analyses of alkaloids, each sample extract was diluted in 1/10 ratio using 1% ammonium acetate buffer. 15 ul of each sample was injected and analyzed on Shimadzu Prominence-i LC-2030C with diode array detector (PDA). HPLC oven temperature was set at 40 °C. Chromatography assay was performed on Kinetex C18 column (150 × 4.6 mm, 5 µm particle size; Phenomenex). Elution was carried out in gradient mode with 1% ammonium acetate buffer (solvent A) and 100% acetonitrile (solvent B). Initially 90:10 gradient ratio of solvent B and solvent A was maintained for 10 min. Eventually, gradient was shifted to 69:31 over 5 min, 10:90 over 2 min, and then 90:10 over 3 min. Eventually, after 18 min ammonium acetate was reduced to 10% and acetonitrile was increased to 90%, continuing till 30 min. HPLC Chromatograms of all standards and plant samples were extracted at 280 nm.

### UPLC-QTOF-MS analysis

The UPLC analysis was performed using a Waters Acquity Ultra-Performance LC system (Waters), equipped with a binary pump system (Waters). An Acquity Ethylene Bridged Hybrid (BEH) C18 column (100 mm_2.1 mm id, 1.7 mm particle size) from Waters was used. The molecules were separated with a mobile phase that consisted of 0.2% acetic acid (solvent A) and 100% acetonitrile (solvent B). The flow-rate was 0.2 mL/min and the gradient elution was initial, 2% B; 0–1 min, 2–100% B; 1–30 min, isocratic 100% B; 30–33 min, 100–2% B; 33–33.5 min, isocratic 2% B; 33–40 min. The MS analyses were carried out on a QTOF Micro mass spectrometer (Waters) equipped with a Z-spray electrospray interface. The analysis was performed in both positive and negative mode and the data were acquired through a mass scan from 100 to 1250 *m/z* without collision. The ionization source parameters were source temperature, 120 °C; cone gas flow rate, 50 L/h and desolvation gas flow rate, 350 L/h; desolvation temperature, 200 °C. The cone and capillary voltages were respectively set at 30 V and 1150 V for the negative mode and 70 V and 1800 V for the positive mode. Nitrogen (99% purity) was used as nebulizing gas. Data acquisition was carried out with the MassLynx 4.1 software. Masses extraction, deconvolution, isotopes and library search was performed using MZMine 2 according to Pluskal *et al*.^66^.

### RNA extraction, next-generation Illumina sequencing, and *de novo* assembly

Five grams of triplicate bulbs, stems, roots, leaves or flowers of *N*. *pseudonarcissus* ‘King Alfred’ were crushed using a pestle and a mortar with liquid nitrogen and transferred to pre-chilled 50 ml tubes to proceed with CTAB (cetrimonium bromide) method for total RNA extraction^34^. After RNA extraction, bulb RNA was selected for Illumina sequencing, and stem, root, leaf and flower RNA were used for RT-qPCR analysis. For transcriptome analysis, integrity of the bulb RNA was checked on a nanodrop and bioanalyzer. Nanodrop quantification yielded 805.68 ng/µl of total RNA with the ratio 260 nm/230 nm of 2.12 and 260 nm/280 nm of 1.91. Bioanalysis of RNA gave a RNA integrity number (RIN) of 8.3 with 28 S/18 S of 1.546656 confirming the RNA quality as pure and intact *i*.*e*. not degraded.

The mRNA were converted into cDNA library and sequenced through Illumina HiSeq. 2000, PE 100 paired ends, at McGill University and Genome Quebec Innovation Centre (Montreal, Canada). Raw paired reads were trimmed from 3′-end and Illumina sequencing adapters were removed, maintaining 50 bps of minimum read length to obtain surviving paired reads. Trimming and clipping were done using Trimmomatic (http://www.usadellab.org/cms/index.php?page=trimmomatic) for quality filtering to have clean reads^67^. *De novo* assembly of clean reads was done using trinity assembler ( https://github.com/trinityrnaseq/trinityrnaseq/wiki). Once surviving pair data were generated, normalization was performed to eliminate redundant reads in datasets without affecting its *Kmer* content. Final obtained unigenes were functionally annotated using trinotate (http://trinotate.github.io/), using the Trinity normalization utility inspired by the diginorm algorithm^68^. Normalized reads were used to assemble the transcriptome using the Trinity assembler^69^. To quantify the gene transcript abundance, the raw RNA-Seq reads were mapped to assembled transcripts applying Bowtie^70^ using default parameters. The gene transcript abundance was calculated as ‘fragments per million mapped reads per kilobase’ (FPKM) using the RSEM package^71^. The genes with extremely low FPKM values *i*.*e*. with a maximum FPKM of less than 1 across two samples, were filtered out before subsequent analysis.

### RT-qPCR analysis

Two micrograms of RNA from different tissues (bulb, root, stem, leaf, and flower) of triplicate plants of *N*. *pseudonarcissus ‘*King Alfred’ were reverse transcribed to form cDNA using oligo dT primers and the Omniscript Reverse Transcription Kit (Qiagen) according to given manufacturer’s protocol. Gene specific primers were designed using Integrated DNA Technology (www.idtdna.com) and Tm Calculator, New England Biolabs (tmcalculator.neb.com), to select suitable annealing temperatures. The sequences for all of the primers used in this study are listed in Supplementary file 4. SensiFAST SYBER Lo-ROX mix (Bioline) was used to prepare a 20 μl reaction containing 1x SensiFAST SYBER Lo-ROX mix, 200 μM of each forward and reverse primer and 3 µl of cDNA sample. Each experiment was performed in triplicate and histone was used as an internal reference gene. Real-time quantitative PCR was performed on CFX Connect Real-Time PCR System (BioRad). PCR conditions for amplification were 95 °C for 3 min, 95 °C for 10 sec and annealing temperature (varies with gene primers, see Supplementary file 4) for 30 sec for 40 cycles. This was followed by a dissociation step (as provided by software) −95 °C for 10 sec, 65 °C for 5 sec and 95 °C for 5 sec. The comparative 2^−ΔΔCt^ method^72^ was used for relative quantification of the gene expression levels.

### Accession numbers

The sequences described in this paper have been deposited in the National Center for Biotechnology Information Sequence Read Archive (https://www.ncbi.nlm.nih.gov/sra/) under the accession number SRR5788585. Gene transcript sequences were deposited in Genbank with the following accession numbers for nucleotide sequences: tyrosine decarboxylase 1 (MF405171), tyrosine decarboxylase 2 (MF405172), phenylalanine ammonia lyse 1 (MF405173), phenylalanine ammonia lyse 2 (MF405174), cinnamate 4-hydroxylase (MF416091), coumarate 3-hydroxylase (MF416092), 4-coumarate-CoA ligase 1 (MF416093), 4-coumarate-CoA ligase 2 (MF416094), hydroxycinnamoyltransferase (MF416095), norbelladine 4′-O-methyltransferase (MF416096), noroxomaritidine synthase 1 (MF416097), noroxomaritidine synthase 2 (MF416098), noroxomaritidine/norcraugsodine reductase (MF416099) and histone (MF405170).

## Electronic supplementary material

Supplementary Information 

Supplementary file 1
