# Supplementary material for: Transcriptome and metabolome profiling of Narcissus pseudonarcissus ‘King Alfred’ reveal components of Amaryllidaceae alkaloid metabolism
Source: Sci Rep. 2017 Dec 11;7:17356. doi: 10.1038/s41598-017-17724-0 (PMC5725579; doi:10.1038/s41598-017-17724-0)
Supplement: Supplementary file 1 — Supplementary Information [file 41598_2017_17724_MOESM1_ESM.pdf]

Transcriptome and metabolome profiling of *Narcissus pseudonarcissus* 'King Alfred' reveal components of Amaryllidaceae alkaloid metabolism.

Aparna Singh[1] and Isabel Desgagné-Penix[1,2]

Supplementary files

Supplementary file 1 List of *m/z* and metabolites identified through UPLC-QTOF-MS analysis in negative and positive ionization mode for different tissues of *N. pseudonarcissus* ‘King Alfred’.

Please see Supplementary Dataset 1 (excel file).

Supplementary file 2 Illumina sequencing output and assembly overview.

**Read Trimming and Clipping of Adapters**	
Raw Paired Reads[a]	73081603
Surviving Paired Reads[b]	66054792
Surviving Paired Reads[c] (%)	90.385
**Normalization**	
Surviving Paired Reads after normalization[d]	10523999
Surviving Paired Reads after normalization[e] (%)	15.932
**Trinity *de novo* Assembly**	
Nb. Transcripts[f]	195347
Nb. Components[f]	98332
Total Transcripts Length (bp)	148818643
Max. Transcript Length (bp)	13507
Min. Transcript Length (bp)	224
Median Transcript Length (bp)	478
Mean Transcript Length (bp)	761
N50 (bp)[g]	1150
**BLAST annotation and filtered annotated components**	
Nb. Transcripts	11708
Nb. Components	4288
Total Transcripts Length (bp)	17131946
Max. Transcript Length (bp)	9315
Min. Transcript Length (bp)	297
Median Transcript Length (bp)	1308
Mean Transcript Length (bp)	1463
N50 (bp)[h]	1768

[a] Number of Paired Reads obtained from the sequencer
[b] Number of Remaining Paired Reads after the trimming step
[c] Percentage of Surviving Paired Reads / Raw Paired Reads
[d] Number of remaining Paired Reads after the normalization step
[e] Percentage of Surviving Paired Reads after normalization / Surviving Paired Reads after trimming
[f] Trinity has created a list of transcripts (contigs) representing the transcriptome isoforms. The transcripts are grouped in components loosely representing genes. Transcript names are prefixed by the component/gene name e.g. transcripts c115_g5_i1 and c115_g5_i2 are derived from the same isolated de Bruijn graph and therefore share the same component/gene number c115_g5.
[g] Corresponding contig length distribution figure N50 = 1150bp below
[h] Corresponding contig length distribution figure N50 = 1768bp below

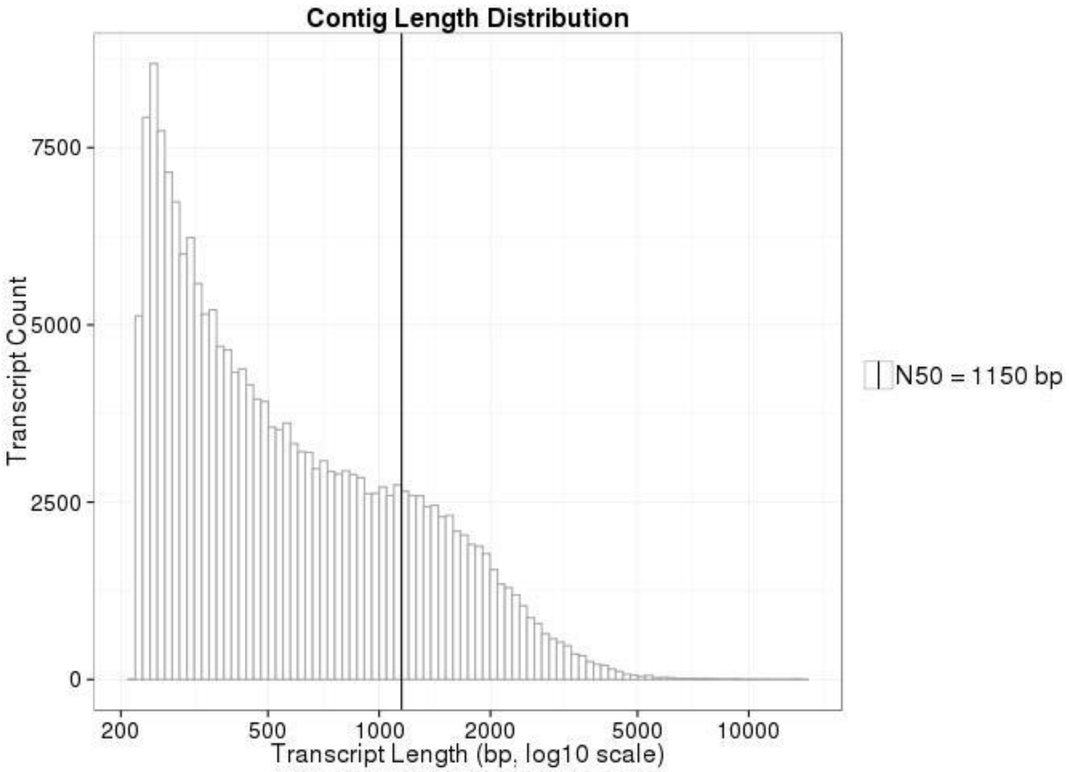
Contig Length Distribution
7500
5000
2500
0
Transcript Count
200
500
1000
2000
5000
10000
Transcript Length (bp, log10 scale)
N50 = 1150 bp

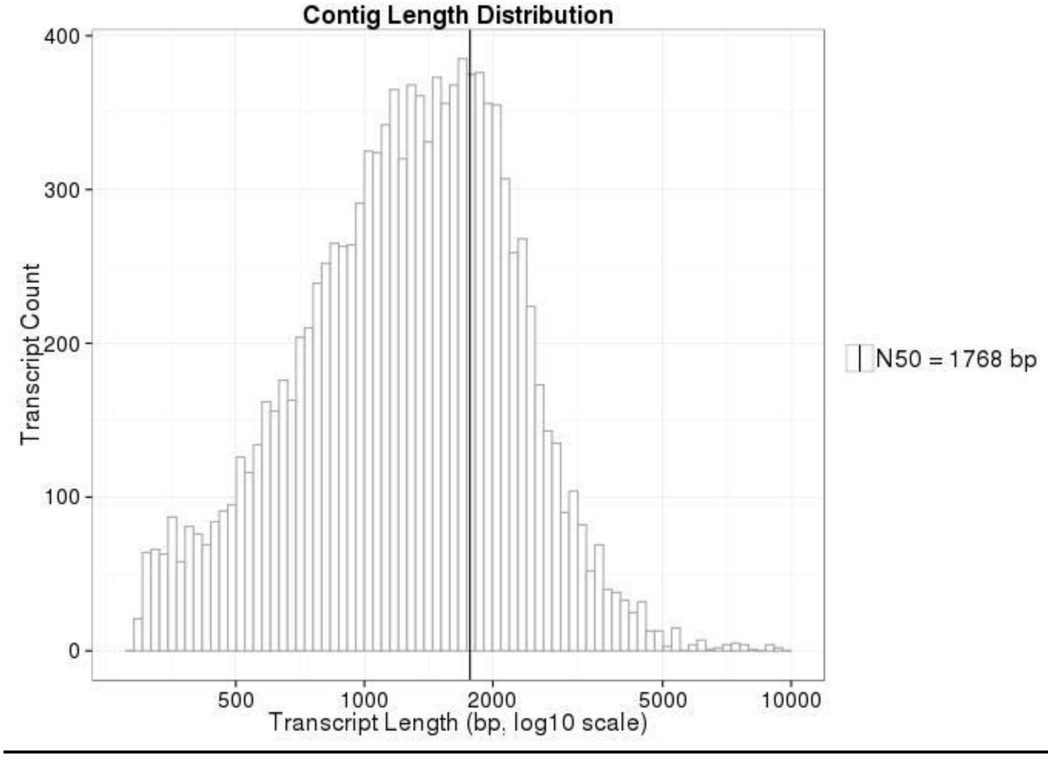
Contig Length Distribution
400
300
200
100
0
Transcript Count
500
1000
2000
5000
10000
Transcript Length (bp, log10 scale)
N50 = 1768 bp

Supplementary file 3 Top 125 most highly expressed gene transcripts with their closest homolog species in the *N. pseudonarcissus* ‘King Alfred’ bulb transcriptome.

Rank	count	Annotation	Species name
1 to 4	5441800	186K protein, protein P1, P2 and 2A	*Narcissus mosaic virus strain New Zealand*
5	229410	metallothionein-like protein	*Arachis hypogaea*
6	206386	peroxidase 42-like	*Eucalyptus grandis*
7	199053	hypothetical protein BVRB_036970	*Beta vulgaris subsp. Vulgaris*
8	139000	sucrose synthase 1-like	*Elaeis guineensis*
9	128069	polyprotein	*Artichoke latent virus*
10	108077	alpha-tubulin	*Moringa oleifera*
11	99080	probable 2-oxoglutarate-dependent dioxygenase At3g49630	*Elaeis guineensis*
12	89648	YTH domain-containing family protein 2-like	*Elaeis guineensis*
13	85872	metallothionein-like protein	*Hyacinthus orientalis*
14	85307	no match	
15	72063	hypothetical protein PRUPE_ppa012400mg	*Prunus persica*
16	69638	Bifunctional 6(G)-fructosyltransferase/2,1-fructan:2,1-fructan 1-fructosyltransferase	*Allium cepa*
17	66209	vacuolar-processing enzyme	*Elaeis guineensis*
18	65737	hypothetical protein CISIN	*Citrus sinensis*
19	65434	(2Fe-2S)-binding protein	*Sulfitobacter donghicola*
20	64944	catalase isozyme A	*Musa acuminata subsp. Malaccensis*
21	64724	hypothetical protein UU56_C0010G0013	*Microgenomates (Curtissbacteria) bacterium*
22	60238	unnamed protein product	*Coffea canephora*
23	60009	ABC transporter F family member 1-like	*Phoenix dactylifera*
24	58301	hypothetical protein OsI_37065	*Oryza sativa Indica Group*
25	55312	glycine-rich RNA-binding protein GRP2A	*Vitis vinifera*
26	54721	glyceraldehyde 3-phosphate dehydrogenase	*Daucus carota*
27	54490	probable aquaporin PIP1-2	*Musa acuminata subsp. Malaccensis*
28	53526	cathepsin B-like	*Musa acuminata subsp. malaccensis*
29	48694	no match	
30	48584	no match	
31	46591	Aspartic proteinase nepenthesin-1 precursor, putative	*Ricinus communis*
32	46472	cleft lip and palate transmembrane protein 1 homolog	*Phoenix dactylifera*

33	46325	ATPase	*Achromobacter sp.*
34	44628	HMG-domain containing protein	*Narcissus pseudonarcissus*
35	44009	SAM-synthetase	*Cicer arietinum*
36	43835	enolase	*Populus euphratica*
37	43141	hypothetical protein FG05_05736	*Fusarium graminearum*
38	42965	probable aquaporin TIP1-1	*Phoenix dactylifera*
39	42852	granule-bound starch synthase 1, chloroplastic/amyloplastic-like	*Elaeis guineensis*
40	42843	adenosylhomocysteinase-like	*Phoenix dactylifera*
41	42692	no match	
42	42304	sucrose synthase 1	*Elaeis guineensis*
43	40806	ADP-ribosylation factor	*Erythranthe guttata*
44	40737	ATP synthase subunit beta, mitochondrial	*Elaeis guineensis*
45	40497	nonspecific lipid transfer protein	*Vitis pseudoreticulata]*
46	40320	elongation factor 2	*Musa acuminata subsp. malaccensis*
47	40094	heat shock protein 90-2	*Tarenaya hassleriana*
48	38591	replication-associated protein A	*Peptoclostridium difficile CD44*
49	34429	uncharacterized protein LOC103702207	*Phoenix dactylifera*
50	34251	60S acidic ribosomal protein P0-like	*Musa acuminata subsp. Malaccensis*
51	34104	DEAD-box ATP-dependent RNA helicase 7-like	*Musa acuminata subsp. Malaccensis*
52	34005	glycine-rich RNA-binding protein GRP2A	*Vitis vinifera*
53	33775	uncharacterized protein LOC105044022	*Elaeis guineensis*
54	33570	no match	
55	33203	vignain-like	*Phoenix dactylifera*
56	32626	UDP-glucuronate 4-epimerase 6	*Vitis vinifera*
57	32278	sucrose 1-fructosyltransferase	*Allium cepa*
58	32251	60S ribosomal protein L3	*Ornithogalum longebracteatum*
59	32108	protease inhibitor/seed storage/lipid transfer protein	*Hyacinthus orientalis*
60	31953	translationally-controlled tumor protein homolog	*Oryza brachyantha*
61	31581	ADP,ATP carrier protein 1, mitochondrial-like	*Malus domestica*
62	31543	S-norcoclaurine synthase 1-like	*Phoenix dactylifera*
63	31433	dimeric mannose specific lectin protein precursor	*Narcissus hybrid cultivar 2*
64	31350	probable mannose-1-phosphate guanylyltransferase 1	*Elaeis guineensis*
65	31335	histone H1	*Zea mays*
66	31269	ruBisCO large subunit-binding protein subunit beta, chloroplastic	*Phoenix dactylifera*
67	31214	GDSL-motif lipase	*Agave americana*
68	30708	MULTISPECIES: hypothetical protein	*bacteria*

69	30518	hypothetical protein M569_09780	*Genlisea aurea*
70	30159	variant of histone H1	*Lilium longiflorum*
71	29602	caffeic acid O-methyltransferase-like protein mRNA, complete cds	*Narcissus tazetta cultivar Huanghua No.2*
72	29577	ADP-ribosylation factor 2 (LOC109851241), mRNA	*Asparagus officinalis*
73	29483	oryzain alpha chain-like (LOC109841121), mRNA	*Asparagus officinalis*
74	29244	elongation factor 1-beta (LOC101493594), mRNA	*Cicer arietinum*
75	29164	ubiquitin-conjugating enzyme mRNA, complete cds	*Lycoris longituba*
76	29109	polyadenylate-binding protein RBP45-like (LOC109833544), mRNA	*Asparagus officinalis*
77	28815	pectin acetylesterase 7-like (LOC109839624), transcript variant X2, mRNA	*Asparagus officinalis*
78	27925	elongation factor 1-alpha (LOC100246711), mRNA	*Vitis vinifera*
79	27903	60S ribosomal protein L5 (LOC109837563), mRNA	*Asparagus officinalis*
80	27631	chitinase-like protein 1 (LOC103992403), mRNA	*Musa acuminata subsp. malaccensis*
81	27532	no match	
82	27365	heat shock protein 90 (HSP90) mRNA, complete cds	*Ornithogalum longebracteatum*
83	27220	heat shock cognate 70 kDa protein 2-like (LOC109848789), mRNA	*Asparagus officinalis*
84	27027	26S proteasome regulatory subunit 4 homolog B (LOC109841322), mRNA	*Asparagus officinalis*
85	26749	putative iron/ascorbate oxidoreductase mRNA mRNA, partial cds	*Narcissus pseudonarcissus*
86	26718	metallothionein-like protein type 2 (LOC110108418), mRNA	*Dendrobium catenatum*
87	26526	calnexin homolog (LOC109824517), mRNA	*Asparagus officinalis*
88	26418	uncharacterized LOC103985898 (LOC103985898), transcript variant X2, ncRNA	*Musa acuminata subsp. malaccensis*
89	26344	myosin-2 heavy chain-like (LOC109819892), transcript variant X2, mRNA	*Asparagus officinalis*
90	26328	xyloglucan endotransglucosylase/hydrolase protein 23 (LOC103976880), mRNA	*Musa acuminata subsp. malaccensis*
91	26162	no match	
92	25959	60S ribosomal protein L10 partial mRNA	*Morus notabilis*
93	25756	methyltransferase PMT26 (LOC109837966), mRNA	*Asparagus officinalis*
94	25549	no match	
95	25507	60S ribosomal protein L6-2-like (LOC109837539), mRNA	*Asparagus officinalis*
96	25268	no match	
97	25216	eukaryotic initiation factor 4A-8 (LOC109828423), mRNA	*Asparagus officinalis*

98	24687	MBK-2015 vittatine 11-hydroxylase mRNA, complete cds	*Galanthus sp.*
99	24672	no match	
100	24436	40S ribosomal protein S6 (LOC109833973), transcript variant X2, mRNA	*Asparagus officinalis*
101	24414	cellulose synthase A catalytic subunit 5 [UDP-forming] (LOC109843790), mRNA	*Asparagus officinalis*
102	24382	soluble starch synthase 2-2, chloroplastic/amyloplastic (LOC109833869), mRNA	*Asparagus officinalis*
103	24107	GTP-binding protein SAR1A (LOC109819697), mRNA	*Asparagus officinalis*
104	23974	heterogeneous nuclear ribonucleoprotein 1-like (LOC109360644), mRNA	*Lupinus angustifolius*
105	23925	no match	
106	23919	thiamine thiazole synthase 2, chloroplastic (LOC107488645), mRNA	*Arachis duranensis*
107	23541	reticulon-like protein B3 (LOC105041675), mRNA	*Elaeis guineensis*
108	23366	histone H2A.4 (LOC103850452), mRNA	*Brassica rapa*
109	23326	alpha-amylase 3, chloroplastic (LOC109820177), transcript variant X4, mRNA	*Asparagus officinalis*
110	23207	60S ribosomal protein L8 (LOC101785583), mRNA	*Setaria italica*
111	23188	no match	
112	23186	V-type proton ATPase subunit c1 (LOC109825900), mRNA	*Asparagus officinalis*
113	23100	aquaporin PIP2-6 (LOC103994286), mRNA	*Musa acuminata subsp. malaccensis*
114	22719	guanine nucleotide-binding protein subunit beta-like protein A (LOC105041173), mRNA	*Elaeis guineensis*
115	22698	putative cysteine proteinase mRNA, partial cds	*Narcissus pseudonarcissus*
116	22585	fructose-bisphosphate aldolase 1, cytoplasmic (LOC109836704), mRNA	*Asparagus officinalis*
117	22513	pyrophosphate-energized vacuolar membrane proton pump-like (LOC109841169), mRNA	*Asparagus officinalis*
118	22463	cytosolic isocitrate dehydrogenase [NADP]-like (LOC109840741), mRNA	*Asparagus officinalis*
119	22356	vacuolar-sorting receptor 1-like (LOC109851365), mRNA	*Asparagus officinalis*
120	22266	serine hydroxymethyltransferase 4-like (LOC103696913), mRNA	*Phoenix dactylifera*
121	22258	coatomer subunit gamma-2 (LOC109833116), mRNA	*Asparagus officinalis*
122	21960	annexin D2-like (LOC109833942), mRNA	*Asparagus officinalis*
123	21777	uncharacterized protein At1g04910-like (LOC109832548), mRNA	*Asparagus officinalis*

124	21747	cellulose synthase A catalytic subunit 1 [UDP-forming] (LOC109828772), mRNA	*Asparagus officinalis*
125	21722	norbelladine 4'-O-methyltransferase (N4OMT3) mRNA, complete cds	*Narcissus aff. pseudonarcissus* MK-2014
sum 125 top	10638280		
sum total surviving reads	66054792		
% top 125	16,1052362		
% virus	8,238312218		

Supplementary file 4 List of real time qPCR primer sequences used in this study.

Gene	Tm	Forward primers (5′-3′)	Reverse primers (5′-3′)	Amplicon size (bp)
TYDC1	56°C	TTGGTGTCGGCGGATGAG	TGCTTCTCCTCCGTGAGG	120
TYDC2	56°C	GTGCCAGGCGTAACCCATTGG	CTGCCCAGAAGACAGGAATTGG	219
PAL1	52.6°C	GAGGAGAATCTGAAGAGC	CATCAAAGGGTAAGTGC	186
PAL2	56°C	CAAGCTGTCAAAAACACGGTG	AAGGGTCGTCGATGTACGTG	148
C4H	53°C	GTATTTGAAGATTTGCAAGC	GCTGCAACATTGATGTTCTC	210
C3H	56°C	CAGGTGCTTCGCCGAGTGG	CCTCACCTTCACGTAGTGGG	226
4CL	57°C	GGCTATTTGAATGACCCTGAGG	CCGAGCTTTTCGACGGAACTAC	277
HCT	53.2°C	CGACGAGGAGTATGTGAGG	CTCCAACGAAACCAGAACC	197
N4OMT	55°C	GTGAAGCTCGTCAGGATTGG	CAGGTTTCTGAAAGAGAGCC	127
CYP96T	57°C	TGCTATGGCGAGGATGAAGG	ACATGTCCCTTCACCATCTG	228
NorRed	57°C	TCCGGGAGCCATAAGAACGC	GGTGATGTAGGAAGCAGATGG	160
His	55°C	GTCTGCCCCAACAACTGGAGG	GCTTCCTAATCAGTAGCTCG	113
